# Social interactions, experiences with adverse life events and depressive symptoms in individuals with visual impairment: a cross-sectional study

**DOI:** 10.1186/s12888-020-02652-7

**Published:** 2020-05-12

**Authors:** Audun Brunes, Trond Heir

**Affiliations:** 1 Section for Trauma, Catastrophes and Forced Migration - Adults and Elderly, Norwegian Centre for Violence and Traumatic Stress Studies, PB 181 Nydalen, Oslo, NO-0409 Norway; 2 Institute of Clinical Medicine, Faculty of Medicine, University of Oslo, PB 1171 Blindern, Oslo, NO-0318 Norway

**Keywords:** Abuse, Blindness, Bullying, Depression, Social isolation, Social support, Visual impairment

## Abstract

**Background:**

Knowledge about the high rates of depression in people with visual impairment (VI) remains unclear. The study aimed to examine whether depressive symptoms in people with VI were associated with social isolation, perceived social support and lifetime exposure to bullying, physical abuse or sexual abuse.

**Methods:**

An anonymous telephone survey was conducted from January to May 2017 in an age-stratified sample of adults with VI who were members of the Norwegian Association of the Blind and Partially Sighted. Participants were asked questions about social isolation, perceived social support, and past experiences with bullying and abuse. Depressive symptoms were measured by the nine-item Patient Health Questionnaire (PHQ-9). We calculated unadjusted and full-adjusted exponentiated beta-values (Exp(β)) and corresponding 95% confidence intervals (CIs) using generalized linear models.

**Results:**

Overall, 736 (61%) adults participated in the study. The mean depression scores were 5.24 (SD: 5.3, range: 0–27), 4.61 for men and 5.77 for women. Results from the full-adjusted model showed higher levels of depressive symptoms among participants who reported social isolation (Exp(β): 1.89, 95% CI: 1.63–2.20), lower levels of perceived social support (Exp(β): 1.55, 95% CI: 1.31–1.83), and past experiences of abuse (Exp(β): 1.41, 95% CI: 1.17–1.70). The strength of the associations between past exposure to bullying or abuse and depressive symptoms was similar for those with low and high levels of support.

**Conclusion:**

Social isolation, perceived support and experiences of adverse events appear to be independently associated with depressive symptoms. Thus, social integration may be appropriate for the promotion of mental health among people with VI.

## Background

Visual impairment (VI) refers to a substantial and often irreversible loss in one or more functions of the visual system [1]. About 253 million people (3.4%) are classified with distance VI at a global basis, of which 36 million are blind and the remaining 217 million have moderate to severe degrees of impairment [2]. Depression is common in people with VI [3–5], and some studies suggest that people with VI have a higher risk of depression than their sighted counterparts. For example, in a registry study from the UK, 18% of older adults with VI had a depressive disorder, against 12% of those without VI [6]. Depression in this population goes often unrecognized and untreated [7], and many of them abstain from seeking professional help due to inaccessible environments and the stigma attached to being visually impaired or blind [7, 8]. A better insight into risk and protective factors of depression in people with VI is important and could be useful for tailoring preventive strategies and professional help to those who need it.

Depression is a multifactorial condition, and its onset and progression are linked to a number of biological, cognitive, social, and environmental factors [9, 10]. We have in previous studies found that visually impaired people are more likely to be socially isolated than people from the general population [11]. We have also reported high rates of bullying and certain traumatic events such as sexual abuse in people with VI [3, 12, 13], and that social withdrawal and isolation are common reactions after traumatic experiences [8, 11]. It is therefore of interest to examine whether poor social interactions and past exposure to bullying and abuse could independently contribute to the high rates of depression in this population.

Two studies of people with VI have included factors of social interactions (social support) and adverse life events in a multifactorial model, with inconsistent results [14, 15]. The first study showed that both lower levels of social support and past-year experiences of adverse life events were associated with current depression [14], though this association was only observed for adverse life events in the other study [15]. In addition, the studies did not include measures of social interactions other than social support. Social interaction is a complex concept, involving elements of social connectedness/isolation, social networks, and perceived and received social support [16–18]. Though often treated interchangeably, these concepts are conceptually and empirically different from each other [17, 18]. While social isolation is related to the quality of the social ties, the concept of social support is more about the availability of social resources [17]. Social networks refers to the structure, size and frequency of social contacts [16].

Studies of the general population shows that perceived social support has both main effects and buffer effects of people’s mental health [19–21]. The buffer hypothesis of social support states that help and care from others is most beneficial during times of crisis or adversities by protecting people against the negative consequences of stress [19, 20]. Social support can be helpful by offering the resources needed for coping; re-establishing hope and meaning; sustaining self-esteem and optimism; changing people’s cognitive appraisal of the problem; and encouraging healthy lifestyle choices [21]. The stress-buffering properties of social support are likely to be restricted to specific sources of support or to different types of stressors [20]. Intentional acts of violence, such as sexual abuse, generally leads to higher levels of stress than that of non-intentional accidents [22], and may thus elicit high support needs among those affected. To our knowledge, no study of people with VI has examined the buffer hypothesis of social support for those who have experienced adverse life events such as bullying or abuse.

Using data obtained from a large, age-stratified sample of people with VI, this study aimed to examine associations of social isolation, perceived social support, and past exposure to bullying or abuse with current levels of depressive symptoms. A second aim was to investigate the extent of which experiences of bullying or abuse was associated with depressive symptoms among those with low or high levels of social support.

## Methods

### Design and participants

This cross-sectional telephone survey included a probability sample of adult members of the Norwegian Association of the Blind and Partially Sighted. All members aged 18 years or older were eligible to participate if they had a diagnosis of VI or progressive eye condition and were able to understand and answer in a Norwegian language. Because most members are of middle age or older, we therefore used age-stratified sampling to allow for more precise estimations across the different age groups. First, the study population was divided into four age groups (years: 18–35, 36–50, 51–65, ≥ 66), and then a separate random sample was taken from each age group. Data were collected between January and May 2017, through structured telephone interviews. The interview guide covered a wide range of topics, including sociodemographic characteristics, cause and nature of vision loss, serious life events, coping, mental health, and quality of life. Each interview took about 30 min to complete.

### Measurements

#### Outcome

In this study, the outcome of interest was depressive symptoms measured by the nine-item Patient Health Questionnaire (PHQ-9) [23]. The validity and utility of the PHQ-9 are good [23], and has often been employed in studies of populations with various chronic conditions [24]. The scale consists of the nine symptoms needed to establish a diagnosis of depressive disorders based on the criteria from the Diagnostic and Statistical Manual of Mental Disorders, fourth edition (DSM-IV). The PHQ-9 also matches the new DSM-V criteria. The participants were asked to report how much a problem had bothered them during the past 2 weeks. The response options for each of the nine items were: ‘*not at all*’ (0), ‘*several days*’ [1], ‘*more than half of the days*’ [2], and ‘*nearly every day*’ [3]. A total score was created by adding each of the nine items together, ranging from 0 to 27 points. The score had a Cronbach’s alpha of 0.84.

#### Social interactions

Social interactions involved measures of social isolation and current levels of social support. Information about the participant’s experiences of social isolation was assessed by a single item from the Three Items Loneliness Questionnaire [25]. The response categories were: ‘*hardly ever*’ (0), ‘*some of the time*’ [1], and ‘*often*’ [2]. For the main analyses, the category ‘some/often’ were compared to the category ‘hardly ever’.

We measured perceived social support by using five items from the Crisis Support Scale (CSS) [26], with the items being: (a) ‘*someone willing to listen*’; (b) ‘*contact with people in similar situation*’; (c) ‘*having someone to talk about thoughts and feelings*’; (d) ‘*sympathy and support from others*’, and (e) ‘*practical help*’. Each item was rated on a seven-point Likert scale, ranging from ‘*never*’ [1] to ‘*always*’ [7]. In this study, the scale had a Cronbach’s alpha of 0.75. We created a social support variable by averaging the ratings to generate a score ranging from 1 to 7 with higher values indicating higher levels of support. The average score was then divided into three equal portions, indicating low (a score < 3), moderate (a score between 3 and 5), and high levels of social support (a score of ≥5). As only 3% of the participants had a score lower than 3, we chose to combine low and moderate levels of support in the present analyses.

#### Adverse life events

A single-item question from the General Nordic Questionnaire for Psychological and Social Factors at Work [27] and two questions from the Life Event Checklist for DSM-5 (LEC-5) [28] were used to query participants about their lifetime exposure to bullying, physical abuse or sexual abuse. Bullying was in this study defined as ‘repeated offensive behaviours over a period of time, in which the person confronted has to experience difficulties defending himself/herself’ [27]. Physical abuse involved violent behaviours such as being attacked, hit, slapped, kicked, or beaten up, whereas sexual abuse comprised rape, attempted rape, and forced sexual acts [28]. An ‘adverse exposure’ variable with three categories was created by combining the responses to the questions about bullying and assaults. Those who responded ‘*no*’ to all three questions were classified as ‘*no bullying and abuse*’. Those who responded ‘*yes*’ to bullying and ‘*no*’ to both physical and sexual assaults were classified into the ‘*bullying, but not abuse*’ category. Those who responded ‘*yes*’ to either physical or sexual assaults irrespective of their reporting on bullying were classified into the ‘*abuse*’ category. In the latter category almost 70% (*n* = 104) of those who had been abused reported past experiences with bullying.

#### Covariates

We identified possible confounding factors based on data availability and previous publications [9, 10, 14, 15]. The suspected confounders of the association between social interactions or adverse exposure and depression were: age (years: 18–35, 36–50, 51–65, ≥ 66), gender, education (years: < 14, ≥ 14), national origin (Norwegian, non-Norwegian), place of residence (village/town (< 20,000 inhabitants), small/large city (≥ 20,000 inhabitants)), marital status (married/cohabitant, unmarried), years since VI onset (congenital, acquired ≥20 years, acquired < 20 years), current status of vision loss (stable, progressive), and having other impairments (no, yes). Moreover, the severity of vision loss was assessed by asking the following question: ‘*How good is your current vision (better-seeing eye, with glasses or contact lenses)*’. The question had the following response alternatives: *‘blind’*, *‘severely impaired’*, *‘moderately impaired’*, and *‘unspecified’*. As only 42 participants reported unspecified VI, we chose to merge the unspecified VI category with the category moderately impaired because we considered those participants to have a lower degree of vision loss than those who reported severe impairment and blindness.

### Statistical methods

All statistical analyses were performed in Stata Version 16 (Stata Corp., Texas, USA). The significance level was set at *p* = 0.05. Descriptive statistics included frequencies, percentages, means and standard deviations (SDs). We also computed mean scores (SDs) of the PHQ-9 across categories of the study characteristics.

Generalized linear models (GLMs) with gamma distribution and log link were used to study associations between perceived isolation, social support, adverse exposure, and current levels of depressive symptoms. The continuous depression variable had a right-skewed distribution. We therefore chose to use the gamma GLM as this model produces good fit to positively skewed outcome data [29]. Results were presented as exponentiated beta-values (Exp β) and corresponding 95% confidence intervals (CIs). The models were either unadjusted or adjusted for covariates plus each of the independent variables. The covariates were either forced into the model (i.e. age, gender, education, marital status and VI severity) or selected based on the best-fitted model principle (i.e. place of residence, years since VI onset, and having other impairments) [30]. Neither national origin nor current status of vision loss improved the model fit and thus the variables were excluded from the model. We also performed some pre-specified subgroup analyses. Because our sample was younger and had a high rate of people with blindness than that of previous studies [4–6, 14, 15], we tested for effect-measure modification according to participant’s age and VI severity. A similar analysis was performed for gender.

We also used gamma GLMs to examine associations between adverse exposure and social support with current levels of depressive symptoms (the buffer hypothesis). We compared exponentiated beta-values for participants with high support levels and those with moderate or low support levels. The analysis was adjusted for age, gender, education, place or residence, marital status, the severity of VI, years since VI onset, and having other impairments. The test for departure from multiplicativity was determined by including a product term of adverse exposure and social support into the regression model [31]. Moreover, we conducted a supplementary analysis to test whether there are certain forms of social support that could serve as better buffers against the stress of experiencing bullying or abuse.

## Results

Of the 1216 members who were contacted, 736 participated (61%) by completing the interviews. Non-participants were more likely than participants to be of young or old age [12]. There were no sources of missing data among the participants; all participants answered all questions and none chose to withdraw from the study.

Table 1 shows the study characteristics and mean depression scores of the VI population. In brief, the mean age of the sample was 51.1 years (SD: 17.2, range: 18–95) and 55% of the participants were women. Forty-five percent had congenital vision loss and the remaining 55% had acquired their VI at some point in life. Of those with acquired vision loss, their mean age of VI onset was 35 years (SD: 20.3, range: 2–76) and almost 90% reported eye disease as the main cause of vision loss. The proportion of the sample experiencing social isolation and low to moderate levels of social support was 41 and 23%, respectively, and nearly half of them reported lifetime exposure to bullying, physical abuse or sexual abuse.

**Table 1:** Study characteristics and mean scores of depressive symptoms in the visual impairment population (*n* = 736)

**Characteristics**	**No. (%) of participants**	**PHQ-9, Mean (SD)**
**Age**		
18–35 years	157 (21.3)	6.00 (5.6)
36–50 years	186 (25.3)	6.72 (6.1)
51–65 years	200 (27.2)	4.54 (4.7)
≥ 66 years	193 (26.2)	3.93 (4.3)
**Gender**		
Women	403 (54.8)	5.77 (5.4)
Men	333 (45.2)	4.61 (5.2)
**Education**		
≥ 14 years	335 (45.5)	4.84 (4.9)
< 14 years	401 (54.5)	5.58 (5.6)
**Place of residence**		
Village/town	399 (54.2)	5.78 (5.6)
Small/large city	337 (45.8)	4.60 (4.9)
**Marital status**		
Married/cohabitant	347 (47.2)	5.57 (5.8)
Unmarried	389 (52.9)	4.87 (4.8)
**VI severity**		
Moderate	254 (34.5)	5.32 (5.3)
Severe	296 (40.2)	5.41 (5.6)
Blind	186 (25.3)	4.86 (5.0)
**Other impairments**		
No	478 (65.0)	4.29 (4.7)
Yes	258 (35.0)	7.00 (5.9)
**Years since VI onset**		
Congenital	345 (46.9)	4.87 (4.9)
Acquired, ≥ 20 years	148 (20.1)	4.30 (4.6)
Acquired, < 20 years	243 (33.0)	6.34 (6.1)
**Social isolation**		
Hardly ever	439 (59.7)	3.41 (3.8)
Some/often	297 (40.3)	7.95 (6.1)
**Social support¤**		
High	567 (77.0)	4.27 (4.4)
Low/moderate	169 (23.0)	8.50 (6.8)
**Adverse exposure**		
None	380 (51.6)	3.87 (4.1)
Bullying only	203 (27.6)	5.46 (5.2)
Abuse	153 (20.8)	8.35 (6.7)

*Notes. PHQ-9* nine-item Patients Health Questionnaire, *VI* visual impairment

This sample had a mean PHQ-9 score of 5.24 (SD: 5.3), and 16% had a score of 10 or greater [24]. With regard to the scoring on the PHQ items, most participants answered positive on the questions about lack of energy (63%), sleeping problems (53%), anhedonia (44%), and depressed mood (41%). Nearly 12 % of the sample reported having thoughts about suicide or self-harm during the past 2 weeks.

### Factors associated with depressive symptoms

The results in Table 2 show unadjusted and adjusted associations of depressive symptoms with factors of social isolation, social support and past experiences with bullying or abuse. In the unadjusted models, all the aforementioned factors were associated with higher levels of depressive symptoms, yielding stronger associations for social isolation (Exp(β) = 2.33) than for lower levels of social support (Exp(β) = 1.99) and past experiences of adverse events (bullying: Exp(β) = 1.41; abuse: Exp(β) = 2.16). In the fully adjusted model, the associations remained significant, despite being drawn fairly towards the null (% decrease: 23–53). In the same model, four out of eight covariates showed statistically significant associations with depressive symptoms, including age, gender, years since VI onset, and having other impairments. None of the subgroup analyses reached statistical significance (*p* > .05).

**Table 2:** Associations between social interaction, exposure to adverse life events and depressive symptoms in the visual impairment population (*n* = 736), unadjusted and fully adjusted for covariates and each of the main independent variables

	**Unadjusted**		**Fully adjusted**	
	**Exp(β) (95% CI)**	***p*-value**	**Exp(β) (95% CI)**	***p*-value**
**Social isolation**				
Hardly ever	1 [Referent]		1 [Referent]	
Some/often	2.33 (2.02–2.70)	**< .001**	1.89 (1.63–2.20)	**< .001**
**Social support**				
High	1 [Referent]		1 [Referent]	
Low/moderate	1.99 (1.69–2.35)	**< .001**	1.55 (1.31–1.83)	**< .001**
**Adverse exposure**				
None	1 [Referent]		1 [Referent]	
Bullying only	1.41 (1.19–1.67)	**< .001**	1.13 (0.95–1.33)	.16
Abuse	2.16 (1.79–2.59)	**< .001**	1.41 (1.17–1.70)	**< .001**
**Covariates**				
**Age (Continuous)**^a^	0.89 (0.85–0.93)	**< .001**	0.91 (0.87–0.95)	**< .001**
**Gender**				
Men	1 [Referent]		1 [Referent]	
Women	1.25 (1.08–1.45)	**.003**	1.32 (1.15–1.51)	**< .001**
**Education**				
≥ 14 years	1 [Referent]		1 [Referent]	
< 14 years	1.15 (1.00–1.34)	.06	1.09 (0.94–1.25)	.26
**Place of residence**				
Village/town	1 [Referent]		1 [Referent]	
Small/large city	0.80 (0.69–0.92)	**.002**	1.08 (0.93–1.24)	.32
**Marital status**				
Married/cohabitant	1 [Referent]		1 [Referent]	
Unmarried	1.14 (0.99–1.32)	.07	0.90 (0.78–1.03)	.14
**VI severity**				
Moderate/other	1 [Referent]		1 [Referent]	
Severe	1.02 (0.86–1.21)	.85	0.92 (0.78–1.08)	.29
Blind	0.91 (0.75–1.11)	.35	0.94 (0.79–1.13)	.51
**Years since VI onset**				
Congenital	1 [Referent]		1 [Referent]	
Acquired, ≥ 20 years	0.88 (0.73–1.07)	.21	0.97 (0.80–1.18)	0.76
Acquired, < 20 years	1.30 (1.10–1.53)	**.002**	1.41 (1.20–1.66)	**< .001**
**Other impairments**				
No	1 [Referent]		1 [Referent]	
Yes	1.63 (1.40–1.90)	**< .001**	1.29 (1.12–1.50)	**.001**

*Notes. CI* confidence interval, *exp* exponentiated, *VI* visual impairment; ^a^: the variable was rescaled into 10-year age intervals

### Buffer hypothesis of social support

We then tested the buffering-properties of social support among participants who had past experiences with bullying or abuse. For participants with low support levels, higher PHQ-9 scores were found among those who have been subjected to bullying only (adjusted Exp(β) = 1.46, 95% CI: 1.06–2.02) and among those who have experienced abuse (adjusted Exp(β) = 1.72, 95% CI: 1.29–2.30), compared with the reference of no past exposure to bullying and abuse. For participants reporting high levels of support, depressive symptoms were associated with abuse (adjusted Exp(β) = 1.57, 95% CI: 1.25–1.97), but not bullying (adjusted Exp(β) = 1.17, 95% CI: 0.96–1.41). Despite heterogeneity of the stratum-specific estimates, the product term did not reach statistical significance (*p* = .70). In addition, we did not find any clear support for our hypothesis that specific forms of support may be more important than others in buffering against the stress that follows bullying or abuse (Table S1, Online Supplement).

## Discussion

In this cross-sectional study of 736 people with VI, we found social isolation, lower levels of social support, and lifetime experiences with bullying, physical abuse or sexual abuse to be independently associated with higher levels of depressive symptoms. In addition, the strength of the associations between adverse exposure and depression was fairly similar for those with low and high levels of perceived social support.

Our findings agrees with the results of Horowitz and colleagues [14] in which both low social support and past exposure to adverse events were independently associated with higher levels of depression. Both our study and that of Horowitz et al. included large samples. However, unlike the study by Horowitz et al. [14], we treated the outcome as a continuous variable, and thus being able to reliably estimate the strengths of the associations and whether they varied across different subgroups of the population. The present study adds to the literature by showing depressive symptoms to be associated with specific types of adverse life events, namely bullying and abuse. The high rates and co-occurrence of bullying and abuse in this population are worrying, and its associations with depressive symptoms support the need of targeted efforts that prevents such events from occurring.

The strong association between social isolation and depression, even after controlling for social support and other factors, illustrate that not only do depression depend on how much care and help people receive from others but it also relates to the closeness of the social ties [17]. Humans have an inherent need of belonging to others. Many people with VI struggle with social interactions [32]. Once people start to avoid or withdraw from social situations this could be the beginning of a downward spiral, resulting in social isolation, loneliness, and depression [33]. Moreover, the social isolation may in part be attributable to the disadvantaged social position of the population. People with VI are more likely to report of discriminating experiences [34]. The lack of enabling environments may force these people into a life of dependency and social deprivation, limiting their access to education, work, and community life [35], and thus having fewer opportunities to meet and connect with others.

Some of the significant covariates needs to be discussed. In particular, we found that young age and female gender were significantly associated with higher levels of depressive symptoms, which is consistent with past research in the general population [36]. Moreover, our data provided further support for the role of having additional impairments as a possible independent risk factor for depression [14, 15, 37]. Depression may develop as people struggle to cope with vision loss and its consequences for daily life [32], and those having other impairments may be confronted with more challenges than those without any impairments.

In this study of people with VI, we found no evidence to support the buffer hypothesis of social support in the aftermath of bullying or abuse. Thus, we failed to confirm findings of some studies from the general population [19–21]. However, bullying or abuse is just one of many challenges that people with VI face in their daily life [3, 32], and our findings may suggest that the benefits of social support in protecting against distress is generally important in this population irrespective of people’s lifetime experiences with bullying or abuse.

### Strengths and limitations

This is one of the first studies addressing social risk factors of depression among people with VI that utilizes a multifactorial approach and that includes a large sample of the population [14, 15]. The use of validated questionnaires, the lack of item non-response and the good response rate increased the validity of the study findings.

The study had some limitations. First, it relied on cross-sectional data, which restricted our ability to make causal inferences about the observed associations. Second, we know little about the possible impact of non-participation and the use of telephone interviews on the estimates. All measures were self-reported and potentially subjected to recall or social desirability bias. Third, the PHQ-9 has been found to be a valid tool for its use in VI populations [24, 38]. However, certain depressive symptoms, and in particular somatic symptoms (e.g., fatigue), bear resemblance to known complications of vision loss [38]. It is therefore somewhat unclear whether the reported problems are clinical features of depression or consequences related to having blindness or low vision. Fourth and lastly, because our sample was recruited from a member organization for the blind and partially sighted, it may be questioned whether it was representative of the broader VI population. Comparisons with official statistics show high comparability on several key factors [39], except that our sample had higher rates of people of young age, blindness, and higher education. Both young age and higher education are well established risk factors of depression [9, 10, 36], which may have affected the level of depression in the study sample.

### Implications

In general, insufficient fulfilling of needs and systematic human rights violations can have substantial consequences on people’s mental health and quality of life, including a higher risk of depression [40]. Our findings of strong associations between social isolation, lower levels of social support, and depressive symptoms suggest that efforts should be made to meet visually impaired people’s needs of social belonging and guaranteeing their fundamental rights of full inclusion and participation in the society. This could be achieved through the creation of safer and more accessible environments [41], by increasing the opportunities for having employment and for starting a family [40, 41], by reducing stigma and discrimination against people with VI [34, 40], as well as through fostering independency and self-reliance of the individual [40].

The high levels of depressive symptoms suggest a need of mental health care. The high levels of depressive symptoms suggest a need of mental health care. It is important that mental health professionals who work with people with VI are aware that many may suffer from social isolation and reactions to past traumas. Both can be central elements in the treatment of depression. Additionally, ophthalmologists and others who provides support and care to people with VI should be well informed about the high risk of depression in this population and consider the need for referral to mental health care. Asking two simple questions about depressed mood and loss of interest in daily activities may be adequate during their evaluation of possible depression [24].

The high prevalence and co-occurrence of bullying and abuse in visually impaired people calls for preventive measures. Prevention strategies should raise public awareness, promote open discussion, and upgrade professional education, service support and guidance [42].

## Conclusions

Our findings of independent associations between various social factors and depressive symptoms among people with VI will be helpful in the design of preventive efforts to reduce the burden of depression in this population. On the basis of these results, preventive programs may include, among other components, strategies that foster social integration of people with VI and reduces their exposure to violence and abuse.

## Supplementary information

**Additional file 1: Table S1.** The association between exposure to adverse events and depression according to participant’s support levels.

## Data Availability

Data are from the research project European Network for Psychosocial Crisis Management – Assisting Disabled in Case of Disaster (EUNAD). Public availability may comprise the privacy of the participants. According to the informed consent given by each participant, the data are to be stored properly and in line with EU Regulation 2017/679 (General Data Protection Regulation (GDPR)). However, anonymized data is available to researchers who provide a methodologically sound proposal in accordance with the informed consent of the participants. Interested researchers can contact project leader Trond Heir (trond.heir@medisin.uio.no) with a request for our study data.

## Abbreviations

CI: Confidence interval
CCS: Crisis Support Scale
Exp: Exponentiated
GLM: Generalized linear models
PHQ-9: Nine-item Patient Health Questionnaire
SD: Standard deviation
VI: Visual impairment
