# Supplementary material for: Social interactions, experiences with adverse life events and depressive symptoms in individuals with visual impairment: a cross-sectional study
Source: BMC Psychiatry. 2020 May 12;20:224. doi: 10.1186/s12888-020-02652-7 (PMC7216619; doi:10.1186/s12888-020-02652-7)
Supplement: Supplementary file 1 — Additional file 1: Table S1. The association between exposure to adverse events and depression according to participant’s support levels. [file 12888_2020_2652_MOESM1_ESM.docx]

**Online supplement**

***The importance of different sources of social support***

**Table S1.** The association between exposure to adverse events and depression according to participant’s support levels.

|  | **Exp(β) (95% CI)^a^** | | |  |
| --- | --- | --- | --- | --- |
| **Social support** | **None (n = 380)** | **Bullying only (n = 203)** | **Assaults (n = 153)** | **Test for EEM, χ2, p-value** |
| **Somebody listening** |  |  |  | 4.0, p=.14 |
| Low-to-moderate | 1 [Referent] | 1.38 [1.11–1.70] | 1.99 [1.57–2.91] |  |
| High | 1 [Referent] | 0.92 [0.69–1.22] | 1.48 [1.10–2.00] |  |
| **Contact with people in similar situations** |  |  |  | 0.3, p=.87 |
| Low-to-moderate | 1 [Referent] | 1.27 [1.04–1.56] | 1.78 [1.43–2.22] |  |
| High | 1 [Referent] | 1.26 [0.91–1.74] | 1.85 [1.32–2.58] |  |
| **Express thoughts and emotions** |  |  |  | 5.2, p=.08 |
| Low-to-moderate | 1 [Referent] | 1.45 [1.16–1.81] | 1.83 [1.43–2.33] |  |
| High | 1 [Referent] | 0.93 [0.72–1.20] | 1.78 [1.35–2.36] |  |
| **Received sympathy** |  |  |  | 5.4, p=.07 |
| Low-to-moderate | 1 [Referent] | 1.48 [1.19–1.83] | 2.12 [1.67–2.70] |  |
| High | 1 [Referent] | 0.88 [0.66–1.16] | 1.54 [1.15–2.05] |  |
| **Practical help** |  |  |  | 1.1, p=.57 |
| Low-to-moderate | 1 [Referent] | 1.35 [1.80–1.69] | 1.86 [1.47–2.35] |  |
| High | 1 [Referent] | 1.12 [0.85–1.46] | 1.77 [1.32–2.37] |  |
